# Combining analytical techniques to assess the translocation of diesel particles across an alveolar tissue barrier *in vitro*

**DOI:** 10.1186/s12989-024-00585-7

**Published:** 2024-05-22

**Authors:** Gowsinth Gunasingam, Ruiwen He, Patricia Taladriz-Blanco, Sandor Balog, Alke Petri-Fink, Barbara Rothen-Rutishauser

**Affiliations:** 1 Adolphe Merkle Institute, University of Fribourg, Chemin des Verdiers 4, Fribourg, 1700 Switzerland; 2 Chemistry Department, University of Fribourg, Chemin du Musée 8, Fribourg, 1700 Switzerland

**Keywords:** Diesel particles (DEPs), Translocation, Transmission electron microscopy (TEM), Ultraviolet – visible (UV-VIS), Lock-in thermography (LIT), A549 cells

## Abstract

**Background:**

During inhalation, airborne particles such as particulate matter ≤ 2.5 μm (PM_2.5_), can deposit and accumulate on the alveolar epithelial tissue. In vivo studies have shown that fractions of PM_2.5_ can cross the alveolar epithelium to blood circulation, reaching secondary organs beyond the lungs. However, approaches to quantify the translocation of particles across the alveolar epithelium in vivo and in vitro are still not well established. In this study, methods to assess the translocation of standard diesel exhaust particles (DEPs) across permeable polyethylene terephthalate (PET) inserts at 0.4, 1, and 3 μm pore sizes were first optimized with transmission electron microscopy (TEM), ultraviolet-visible spectroscopy (UV-VIS), and lock-in thermography (LIT), which were then applied to study the translocation of DEPs across human alveolar epithelial type II (A549) cells. A549 cells that grew on the membrane (pore size: 3 μm) in inserts were exposed to DEPs at different concentrations from 0 to 80 µg.mL^− 1^ ( 0 to 44 µg.cm^− 2^) for 24 h. After exposure, the basal fraction was collected and then analyzed by combining qualitative (TEM) and quantitative (UV-VIS and LIT) techniques to assess the translocated fraction of the DEPs across the alveolar epithelium in vitro.

**Results:**

We could detect the translocated fraction of DEPs across the PET membranes with 3 μm pore sizes and without cells by TEM analysis, and determine the percentage of translocation at approximatively 37% by UV-VIS (LOD: 1.92 µg.mL^− 1^) and 75% by LIT (LOD: 0.20 µg.cm^− 2^). In the presence of cells, the percentage of DEPs translocation across the alveolar tissue was determined around 1% at 20 and 40 µg.mL^− 1^ (11 and 22 µg.cm^− 2^), and no particles were detected at higher and lower concentrations. Interestingly, simultaneous exposure of A549 cells to DEPs and EDTA can increase the translocation of DEPs in the basal fraction.

**Conclusion:**

We propose a combination of analytical techniques to assess the translocation of DEPs across lung tissues. Our results reveal a low percentage of translocation of DEPs across alveolar epithelial tissue in vitro and they correspond to in vivo findings. The combination approach can be applied to any traffic-generated particles, thus enabling us to understand their involvement in public health.

**Supplementary Information:**

The online version contains supplementary material available at 10.1186/s12989-024-00585-7.

## Background

It is now known that air pollution is one of the important risk factors for increasing morbidity and mortality upon short- or long-term exposure [1, 2]. The composition of air pollution is a complex mixture of gases, mists, and particles, and it varies according to source, location, and season [3]. The International Agency for Research on Cancer (IARC) considers outdoor air pollution as one of the major environmental causes of cancer deaths. It has classified particulate matter (PM) as carcinogenic to humans (Group 1) [4, 5]. Air quality standards are defined for air pollutants such as PM_10_ and PM_2.5_, particles with an average aerodynamic diameter of 10–2.5 μm, respectively, to limit their level in the ambient air for environmental and health protection. According to the new World Health Organization guideline published in 2021 [6], their annual mean concentrations should be lower than 15 µg.m^-3^ and 5 µg.m^-3^ for PM_10_ and PM_2.5_, respectively, national air quality standards still exceed these values, highlighting the need for further action on emission sources.

Vehicle emissions, such as from diesel engines contribute to particles released in traffic areas [7]. Diesel exhaust particles (DEPs) are made of elemental and organic carbon in association with metal and metal-oxides originating from lubrication and fuel additives and from engine wear [8, 9]. The role of organic compounds such as polycyclic aromatics hydrocarbons (PAHs), classified as carcinogens Group 1 by the IARC, is carefully studied and considered as important in adverse effects [10]. Depending on their hydrodynamic diameter, inhaled particles are deposited from the nose to the alveoli [11]. The fine particles, i.e., PM_2.5_, reaching the alveolar region can deposit and accumulate on the alveolar lining fluid and epithelial tissue [12, 13]. However, continuous exposure to diesel particles can alter the epithelium [14, 15]. Inflammatory and genotoxicity evidences have been demonstrated within alveolar epithelial in vitro tissue upon exposure to PM [16–19]. Increase in mucus production, a key feature of acute inflammatory and chronic obstructive pulmonary diseases, and translocation events detected by ICP-MS have been both reported within bronchial epithelial in vitro tissue upon exposure to brake wear nanoparticles [20]. The translocation of inhaled particles across the lung and their distribution to other organs such as the brain, heart, liver, and kidney have been highlighted [21]. In rodents’ lungs, inhaled carbon aggregates with a small fraction of radiolabeled Iridium (^192^Ir) were found at low amounts in secondary target organs after 24 h, confirming the translocation and accumulation of inhaled particles in organs beyond the lungs [22]. Similarly, a limited amount of inhaled technetium-99 m (99mTc)-labeled carbon nanoparticles (Technegas) translocated from the human lungs into the systemic circulation, however, the potential effects of few translocated particles were doubted [23–25]. Harmful effects of the ambient particulates on the secondary tissues are progressively elucidated [26, 27], however, there is no strong evidence showing the effects on the secondary tissues are directly caused by the translocated fraction of fine particles. To understand the role of airborne PM on the secondary tissues, its translocation across the air-blood barrier has to be investigated.

Optical methods such as aethalometers or thermal methods are well-accepted tools for measuring elemental carbon (EC) [28, 29]. In Europe, a standardized thermal-optical protocol for measuring atmospheric organic and elemental carbon has been defined and applied worldwide [30]. In this study, we aimed to develop a methodical approach to assess the translocation of DEPs to understand whether these particles could translocate across an alveolar epithelial barrier. For this purpose, absorbance spectroscopy and thermography methods were chosen based on the optical properties of DEPs and optimized in order to assess translocation across permeable cell culture inserts. Thereafter, the optimized methods were applied to assess translocation across alveolar epithelial type II (A549) cells.

The cells were grown in a 2-compartment chamber (Transwell^™^) until they reach high confluency. This enables us to investigate the direct effects of standard reference material 2975 (SRM2975) DEPs collected from forklift diesel engine on the cells. The biological endpoints such as tissue integrity, and cell viability, will be evaluated by measuring the transepithelial electrical resistance (TEER) of the confluent A549 tissue [31], and the lactate dehydrogenase (LDH) release [32] respectively. In addition, the translocation of DEPs across the alveolar epithelial cells will be assessed based on the carbon black properties, i.e., the optical absorption by ultraviolet-visible (UV-VIS) spectroscopy and thermal emission upon heating by lock-in thermography (LIT) techniques.

## Results

### Characterization of DEPs in cell culture medium

Two stock solutions of 2.56 mg.mL^-1^ DEPs were prepared in MQ water, one of them contained 0.05% w/v of pulmonary surfactant, and were both diluted either in MQ water or CCM. In the following descriptions, the terms ‘water-DEPs’ and ‘pulmonary surfactant-DEPs’ are only used to distinguish the stock solutions without and with Curosurf, respectively.

**Fig. 1:**
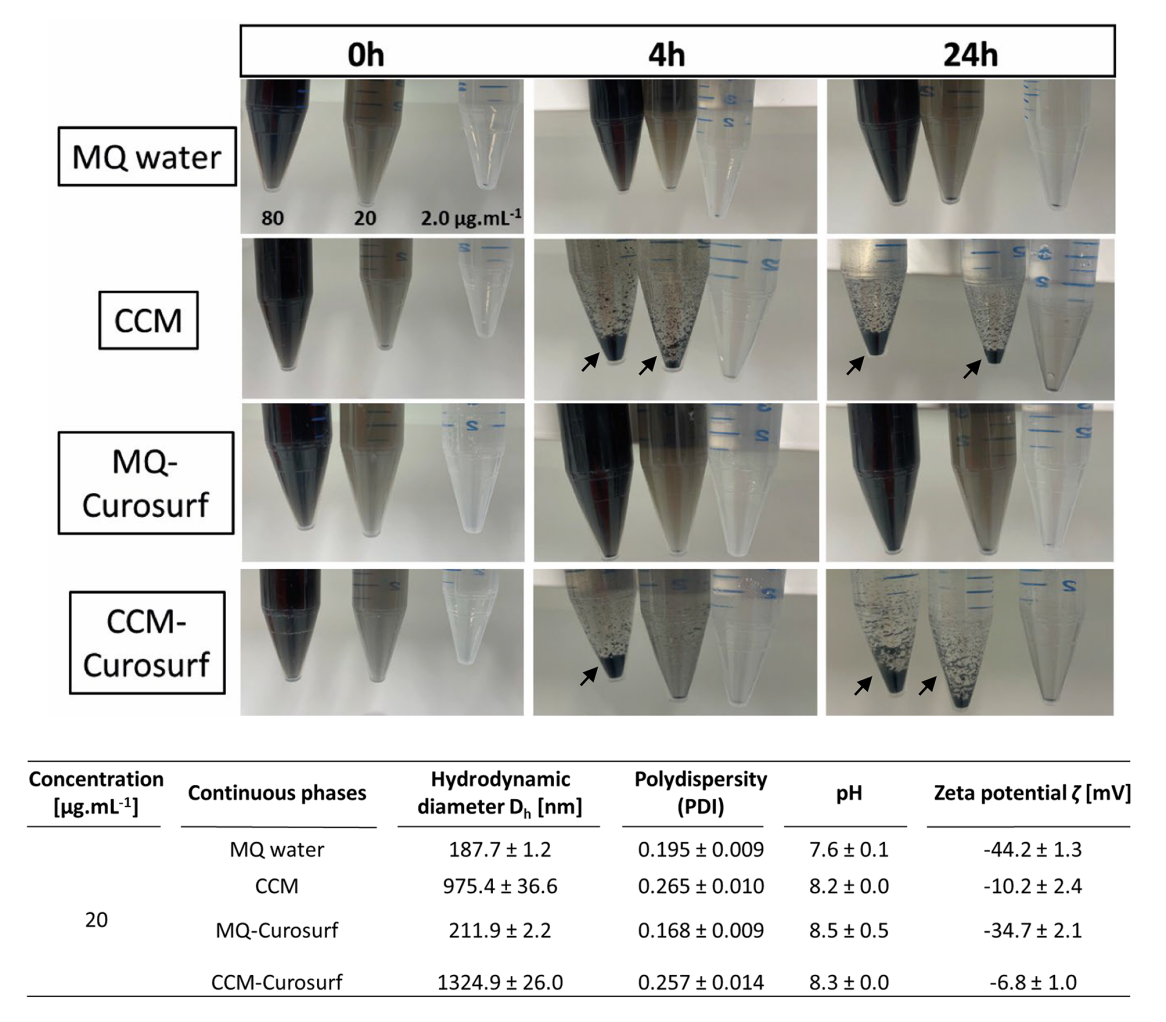
**Characterization of diesel exhaust particles (DEPs, SRM2975) in water-based dispersion and in cell culture medium.** DEPs dispersion in Milli Q (MQ) water, MQ water containing surfactant (MQ-Curosurf), cell culture medium (CCM) and CCM containing surfactant (CCM-Curosurf) at 80, 20 and 2.0 µg. mL^-1^ at 37 °C over time. Agglomerates are indicated with arrows. The physical characteristics including hydrodynamic diameter (D_h_), polydispersity, zeta potential (ζ) and pH of 20 µg. mL^-1^ DEPs in MQ water, MQ-Curosurf, CCM and CCM-Curosurf at 0 h, 37 °C are displayed in the table. The means ± SD from three independent batches were used as technical replicates (*n* = 3). * represents (*p* < 0.05), ** (*p* < 0.01), *** (*p* < 0.001), **** (*p* < 0.0001)

As shown in Fig. 1, water-DEPs at 20 and 80 µg.mL^-1^ showed more sedimentation at the bottom of the 15 mL falcon tubes in cell culture medium (CCM) compared to in water-based dispersion (MQ water) at both time points 4 and 24 h of incubation at 37 °C. Analysis with the Brookhaven 90Plus at time point 0 h of incubation revealed that DEPs are polydisperse (PDI > 0.1) in both MQ water and CCM. The average hydrodynamic diameter $\left({\text{D}}_{\text{h}}\right)$ of DEPs in CCM is 975 nm and is five times higher than in aqueous dispersion with the size of 188 nm (*p* < 0.0001), indicating the formation of bigger DEP agglomerates in CCM. Since inhaled fine particles reach the alveoli region, they will first face the lung lining fluid containing surfactants, therefore pulmonary surfactant-DEPs were also compared in MQ water and CCM (Fig. 1). In terms of sedimentation, there are no differences between water- and pulmonary surfactant-DEPs in MQ water. However, the hydrodynamic diameter and the zeta potential (ζ) of DEPs slightly increased in the presence of surfactant in both MQ water (${\text{D}}_{\text{h}}$: *p* < 0.0001, ζ: *p* < 0.01) and CCM (${\text{D}}_{\text{h}}$: *p* < 0.001, ζ: *p* > 0.05) (Fig. 1). The contributions of the Curosurf and the media to the size and zeta potential data were neglectable, since the scattering signals were weak, and the zeta potential data was in the range of -4 to + 2 mV. Therefore, to mimic the physiological contact in the lungs, further experiments were performed with pulmonary surfactant-DEPs in CCM.

### Microscopic visualization of pulmonary surfactant-DEPs

**Fig. 2:**
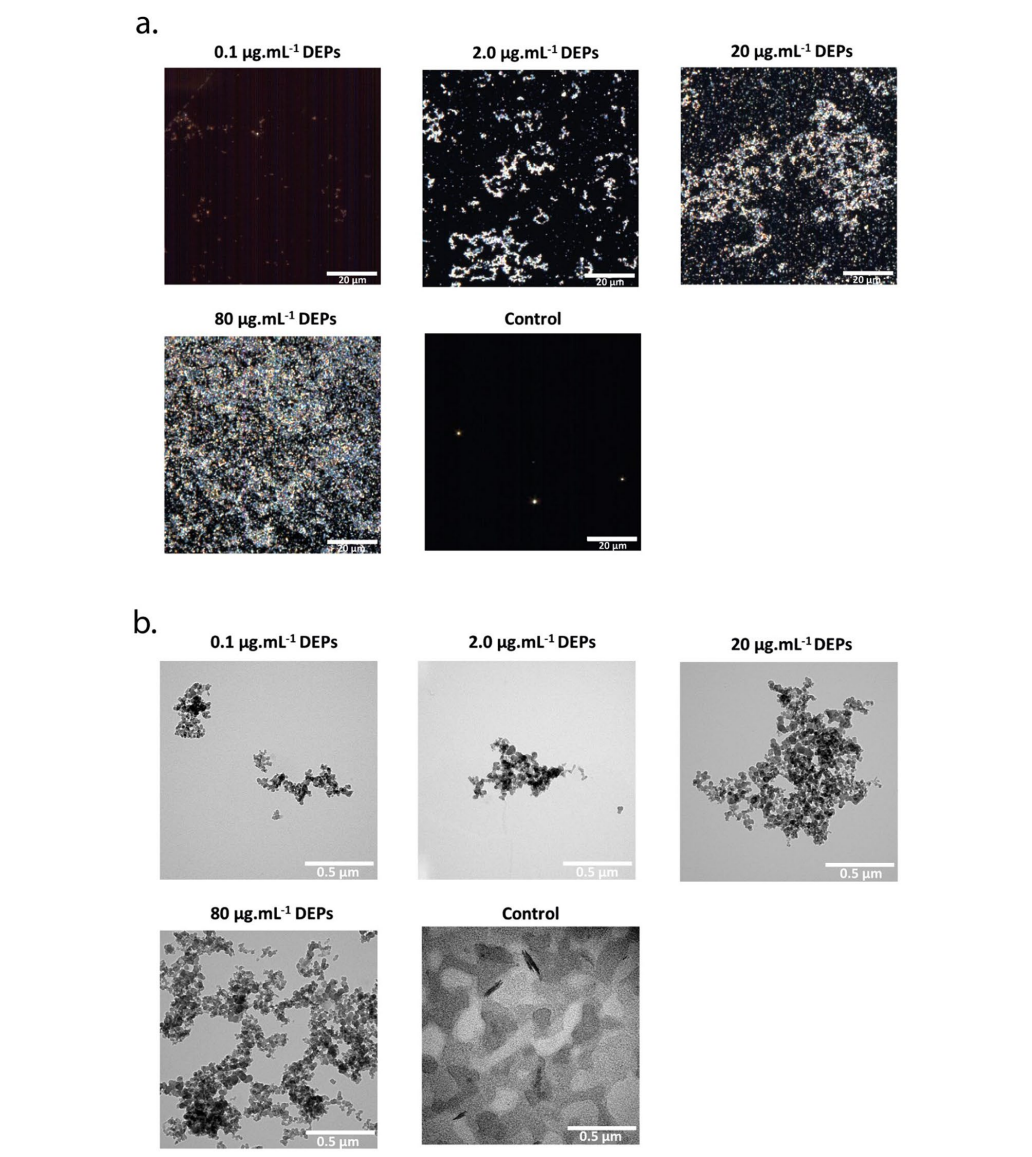
**Visualization of dried DEPs from MQ-Curosurf solution at 0.1, 2.0, 20, and 80 µg.mL**^**-1**^**.** **a**. Enhanced darkfield hyperspectral imaging (EDF-HSI), scale bar: 20 μm, and **b**. Transmission electron microscope (TEM). The control corresponds to the MQ-Curosurf solution without DEPs, scale bar: 0.5 μm

The pulmonary surfactant-DEPs were visualized with enhanced darkfield hyperspectral imaging (EDF-HSI) and transmission electron microscopy (TEM). DEPs ranging from 2.0 to 80 µg.mL^-1^ are detected by the EDF-HSI, however, the visualization is challenging at a very low concentration of 0.1 µg. mL^-1^ (Fig. 2a). While by TEM, the DEPs are visualized at all concentrations (Fig. 2b). These observations highlight the superiority of the TEM over the EDF-HSI to visualize the pulmonary surfactant-DEPs, especially at low concentrations.

### Detection of the translocated fraction of DEPs across porous PET membrane

**Fig. 3:**
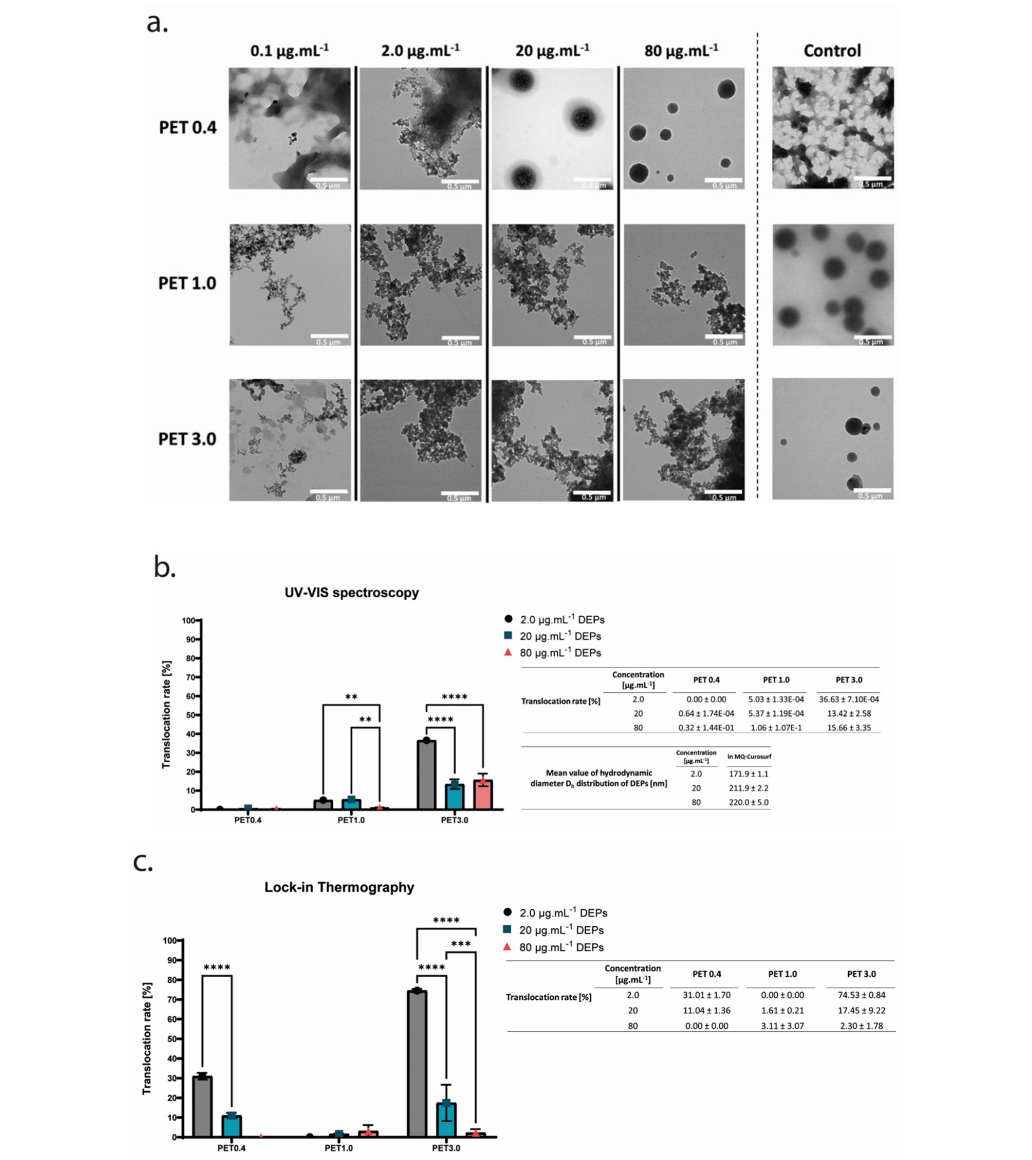
**Visualization and quantification of translocated DEPs into the basal fraction across porous PET membranes. (a)** TEM images of the translocated DEPs across 0.4, 1.0, and 3.0 μm porous membrane collected from the basal cell culture medium. The applied concentrations of the DEPs on the apical side of the membrane are 0, 0.1, 2.0, 20, and 80 µg. mL^-1^ in MQ-Curosurf solution, scale bar: 0.5 μm. **(b)** and **(c)** Percentage of translocation of DEPs across 0.4, 1.0, and 3.0 μm porous membrane determined with ultraviolet-visible (UV-VIS) absorbance spectroscopy **(b)** and lock-in thermography (LIT) **(c)**. The means ± SD from three independent experiments were used as technical replicates (*n* = 3). ** represents (*p* < 0.01), *** (*p* < 0.001), **** (*p* < 0.0001)

Different methods were used to assess the translocated fraction of pulmonary surfactant-DEPs across the apical to basal compartment through porous PET membranes with different pore sizes (0.4, 1.0, or 3.0 μm). By TEM a translocation of DEPs across PET membranes with 1.0 and 3.0 μm pore size was observed at all applied concentrations. For the smallest pore size, i.e. 0.4 μm, a translocation was only detected for the lower concentrations, i.e. DEPs at 2 and 0.1 µg.mL^− 1^ with eventually a trace of the surfactant. Furthermore, absorption spectroscopy (UV-VIS) and thermal emission lock-in-thermography (LIT) were also used to quantitatively determine the translocation of DEPs across the PET membrane with a neglectable contribution from the surfactant (Supplementary S2 and S4). Standard calibration curves with known mass concentrations were successfully established for UV-VIS and LIT (R^2^ = 0.99 and R^2^ = 0.97, respectively) in order to determine the translocation of particles (Supplementary S2). In addition, the limits of detection (LOD) were also calculated for both methods [33]; for UV-VIS, the LOD is 1.92 µg.mL^− 1^ in MQ-Curosurf and 6.60 µg.mL^− 1^ in CCM-Curosurf; whereas for LIT, the LOD is 0.20 µg.cm^− 2^ in MQ-Curosurf (Supplementary S2). Thus, the minimal mass that can be detected and quantified is 1.92 µg with the UV-VIS and 0.22 µg with the LIT from the MQ-Curosurf solution (Supplementary S2) and analysis for the lowest concentration (0.1 µg.ml^− 1^) was not possible. For PET 0.4 μm pore size, the percentage of DEPs translocation that was quantified by UV-VIS is low (0.64% with 20 µg.mL^− 1^ DEPs and 0.32% with 80 µg.mL^− 1^ DEPs) or null (with 2 µg.mL^− 1^). However, by LIT, the percentages are higher (31.01% with 2 µg.mL^− 1^ DEPs and 11.04% with 20 µg.mL^− 1^ DEPs) or null (with 80 µg.mL^− 1^). For PET 1.0 μm pore size, the percentage of translocation by UV-VIS have slightly increased (5.03% with 2 µg.mL^− 1^, 5.37% with 20 µg.mL^− 1^, and 1.06% with 80 µg.mL^− 1^ DEPs) compared to the percent across the PET 0.4 μm pore size, and are comparable with the LIT data (0.00% with 2 µg.mL^− 1^, 1.61% with 20 µg.mL^− 1^, and 3.11% with 80 µg.mL^− 1^ DEPs) ; For PET 3.0 μm pore size, we observed the highest translocation of the DEPs fraction (UV-VIS: 36.63% with 2 µg.mL^− 1^, 13.42% with 20 µg.mL^− 1^, and 15.66% with 80 µg.mL^− 1^ DEPs, LIT: 74.53% with 2 µg.mL^− 1^, 17.45% with 20 µg.mL^− 1^, and 2.30% with 80 µg.mL^− 1^ DEPs) compared to the fraction across PET 0.4 and PET 1.0 μm pore size. Interestingly, both UV-VIS and LIT data indicate that the lowest mass concentration of DEPs (2 µg. mL^− 1^) applied on the apical side shows the highest translocation percentage of DEPs (37% with UV-VIS and 75% with LIT) across the largest pore size (3.0 μm) compared to the middle (20 µg. mL^− 1^) and highest mass concentrations (80 µg. mL^− 1^) Fig. 3.

### The biological effect of the deposited fraction of DEPs on the A549 monolayer

**Fig. 4:**
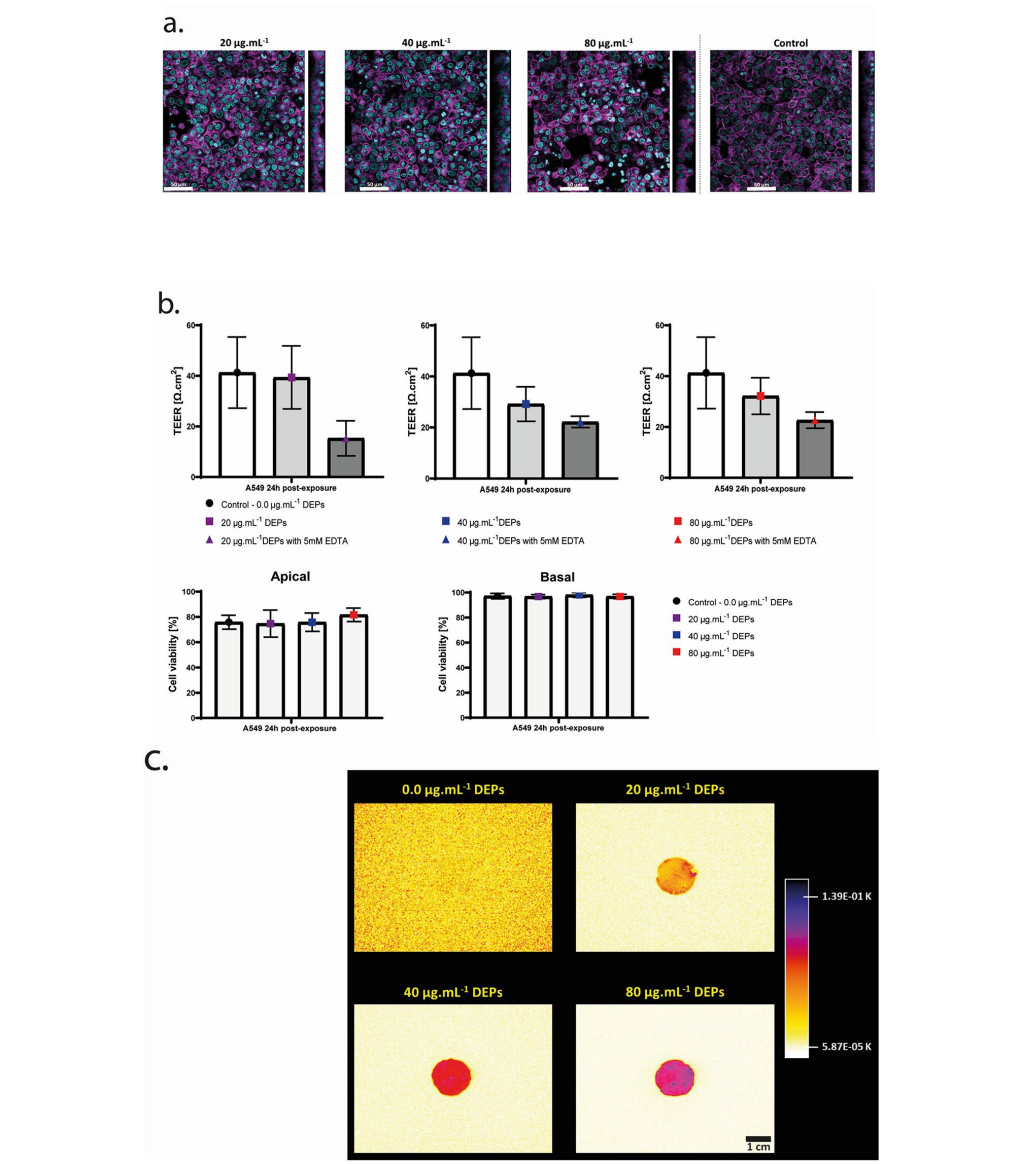
**Effects of deposited DEPs on A549 alveolar cells at 24 h post-exposure. a.** 3D – confocal laser scanning images of deposited DEPs at 20, 40, and 80 µg.mL^-1^ on the A549 monolayer, scale bar: 50 μm. Cell nuclei and F-actin cytoskeleton are stained with DAPI (cyan) and Phalloidin (magenta), respectively. **b**. A549 monolayer integrity is assessed with transepithelial electrical resistance (TEER) measurement and 5 mM of ethylenediaminetetraacetic acid (EDTA) as a positive control. The viability of the A549 cells is evaluated with the release rate of cytosolic lactate dehydrogenase (LDH) in the apical and basal fractions. The means ± SD of three independent experiments performed in duplicates are displayed (*N* = 3). **c**. Thermal emission of deposited DEPs at 20, 40, and 80 µg.mL^-1^ on the A549 monolayer determined with the LIT, scale bar: 1 cm. * represents (*p* < 0.05), ** (*p* < 0.01), *** (*p* < 0.001), **** (*p* < 0.0001)

Based on the obtained results, the highest percentage of translocation of DEPs fraction was measured with the 3.0 μm porous PET membrane. Therefore, A549 cells were grown on the PET membranes with 3.0 μm pores size for 5 days. Moreover, the range of the apical applied concentrations of DEPs has been adapted according to the LODs values of both UV-VIS and LIT methods, hence the cells were exposed to DEPs at 20 µg.mL^-1^ (11 µg.cm^-2^), 40 µg.mL^-1^ (22 µg.cm^-2^), and 80 µg.mL^-1^ (44 µg.cm^-2^) for 4-hours and post-exposed for additional 20-hours after removing the apical DEPs suspension (Fig. 4). After the 20-hours post-exposure, A549 cells were observed under phase contrast microscopy (Supplementary S3), and then stained for immunofluorescence imaging. The 3D-cofocal laser scanning images show a high confluency and an intact cell morphology of the cells before and after exposure to DEPs. The presence of DEPs on the tissue does not affect the TEER values and the cell viability compared to the control after the 24-h post-incubation. However, upon simultaneous exposure to DEPs and 5 mM EDTA, a slight decrease in the TEER measurements is observed at all applied doses compared to the EDTA untreated A549 monolayer control (Fig. 4b). Additionally, the porous membrane of the inserts with fixed cells was cut out and measured with the LIT techniques. As shown in Fig. 4c, DEPs were deposited homogeneously and increased with the applied concentration on the A549 cell layer.

### Detection of the translocated fraction of DEPs across the alveolar epithelial type II (A549) cells grown on 3.0 μm porous PET membrane

**Fig. 5:**
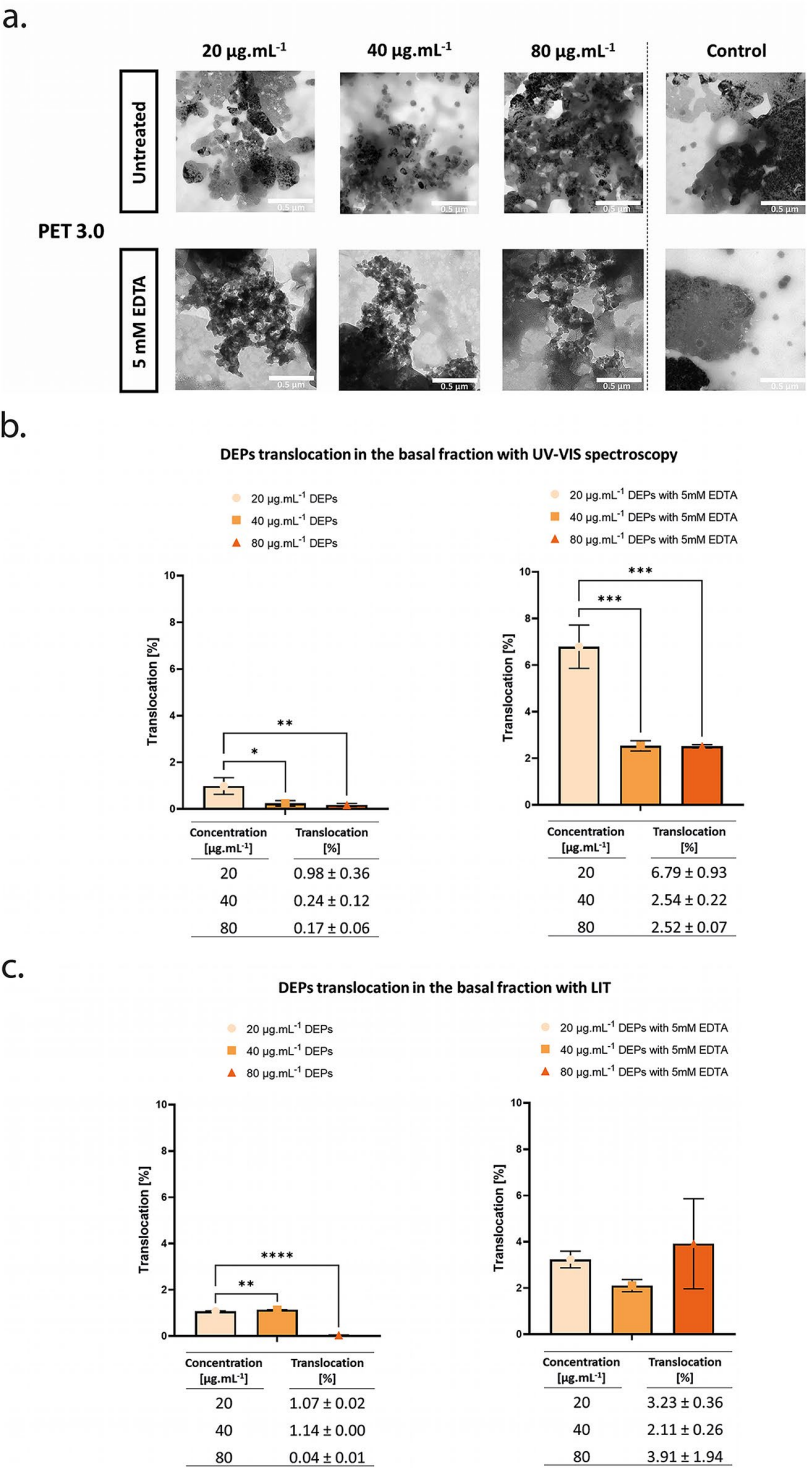
**Visualization and quantification of translocated DEPs across the alveolar epithelial type II (A549) cells grown on 3.0 μm porous PET membrane into the basal fraction. (a)** TEM images of the translocated DEPs across the A549 monolayer in the basal fraction. The applied concentrations on the apical side of the membrane are 20, 40 and 80 µg. mL^-1^ in CCM-Curosurf solution, with and without 5mM EDTA treatment, scale bar: 0.5 μm. **(b) and (c)** The percentage of translocation of DEPs across the A549 monolayer was determined with UV-VIS absorbance spectroscopy **(b)** and LIT **(c)** techniques. The means ± SD of three independent experiments performed in duplicates are displayed (*N* = 3)

The translocated fraction of DEPs across A549 cells was measured with TEM, UV-VIS, and LIT. According to TEM micrographs, the translocated fraction of DEPs was quasi-undetectable because of the unspecific background from the cell culture media and the Curosurf, however, the percentage of DEPs translocation across the tissue was around 1% with both UV-VIS and LIT techniques at the lowest concentration (20 µg.mL^-1^). Interestingly, at the highest apically applied concentration (80 µg.mL^-1^) the percentage of translocation is almost zero. The translocated fraction is successfully visualized by TEM following exposure of A549 layer to DEPs and 5 mM EDTA (Fig. 5a). The simultaneous exposure has increased the percentage of DEPs translocation at all applied concentrations measured by UV-VIS and LIT (Fig. 5b and c). Surprisingly, UV-VIS data show the highest percentage of DEPs (7%) with the lowest concentration with EDTA (20 µg.mL^-1^) compared to the middle (40 µg. mL^-1^) and highest mass concentrations (80 µg. mL^-1^) with EDTA.

## Discussion

Particulate matter such as PM_10_ and PM_2.5_ are anthropogenic and constantly released in the coastal, urban traffic areas, and in the occupational workplace such as airports. The population living close to the sources is daily exposed to the traffic-borne particles, a mixture of PM and chemical substances. PM_2.5_ are known to reach the alveoli region upon inhalation and translocate across the air-blood barrier (brain and lungs). The translocated fraction of DEPs can directly enter the blood circulation to reach the other organs such as liver, intestine, and urinary bladder to induce adverse health effects. However, the fraction of translocation of traffic-related particles is poorly understood due to the lack of analytical methods. Therefore, we present in this study a methodical approach to assess the translocation fraction of traffic-borne particles across the alveolar epithelial in vitro tissue.

### Pulmonary surfactant-coated DEPs in cell culture medium

In our study, we tested the translocation of DEPs across the alveolar tissue. Therefore Curosurf, a pulmonary surfactant collected from porcine lungs, was used to mimic the interaction of biological fluids with the particles upon interaction with the lung surface [34]. The physicochemical properties of DEP with the surrounding environments is important to consider for in vitro studies [35]. Therefore, the behavior of the pulmonary surfactant-DEPs was investigated in different solution (MQ-water and CCM) at 37 °C. Fraction of pulmonary surfactant-DEPs was observed to agglomerate and sediment in the CCM as predicted in the Derjaguin-Landau-Verwey-Overbeek (DLVO) theory [36]. Since the bio-macromolecules and the increasing salt concentration in CCM influence the agglomeration state of DEP compared to in MQ-water. Amino acids with hydrophobic and positively charged residues, as well as vitamins from the CCM, can adsorbed with the surfactant on the surface of particles, thus increasing the size of particles (protein corona). Moreover, the increase of the ionic strength in the CCM suppresses the electrostatic repulsion between particles, thus reducing the Debye-length and increasing the collapsing of the stern layer to form agglomerates [35, 37]. Since pulmonary surfactant-DEPs agglomerate and sediment over time in CCM, the physical characterization of DEPs was carried out at time point 0 h of incubation at 37 °C after sonication, and the duration of exposures of DEPs on empty inserts and on A549 cells was done for only 4 h to allow the contact between porous PET membrane - DEPs, and cells - DEPs.

### Microscopic visualization of pulmonary surfactant-DEPs

The visualization of dried pulmonary surfactant-DEPs by light and electron microscopy showed the formation of agglomerates. Drying of DEPs increases the compaction of the material and contributes as well with the adsorbents to the light scattering phenomena. However, the visualization of DEPs with the EDF-HSI is limited to 0.1 µg.mL^-1^ with a low resolution (half of the wavelength). However, we can detect the particles, create a library of spectra characterizing them, and identifying them on biological samples with cells (Supplementary S1), but then there is a need to go to the TEM a high-resolution technique, for visualization of DEPs at lower concentrations, as well as for an ultrastructural analysis. The TEM can provide more informative images with the higher resolution for DEPs visualization compared to the EDF-HSI in this study. The clear visualization of the pulmonary surfactant-DEPs under TEM is likely due to the high compaction degree of carbons after drying, thus improving the contrast of the sample and enabling the determination of the physical properties such as fractal dimension and the size of the primary core of black carbon aggregates [38], in addition to a potential role of the surfactant upon translocation of DEPs across a lung tissue. Therefore, we used TEM to visualize the translocated fraction, and the EDF-HSI could be used to observe the deposited fraction of pulmonary surfactant-DEPs within biological samples.

### Detection of the translocated fraction of DEPs across porous PET membrane and estimation of the translocation ratio of DEPs with UV-VIS and lock-in thermography methods

TEM analysis enables to detect qualitatively the translocated fraction of DEPs, however, to quantify the translocation fraction across a physical barrier is not sufficient. Traffic-borne particles such as DEPs are released in the ambient air. They are usually characterized by a nanoparticle sizer, a Scanning Mobility Particle Sizer (SMPS) spectrometer [39] and by electron microscopies (SEM/TEM) for the morphology [9, 40]. Another method to detect soot particles is by an Aethalometer, an optical measurement of light absorbing carbonaceous aerosols [41]. Moreover, the quality of the air can be analyzed using infrared geostationary remote sensing to determine PM ground-level composition and concentration [42]. Based on those applied methods, in this study, UV-VIS and LIT are optimal instruments to detect quantitatively the mass of carbon particulates in the basal fraction. As the particulates are black, we assume that the absorbance predominates the optical attenuation of the DEPs in the solution. Furthermore, as the material is heat-conductive, we also assume that repeated LED exposures should homogenize the heat in the entire material and gives the best thermal signal from the dried sample. Thus, the absorbance and the thermal emission of DEPs were tested for a linear correlation to the mass-based concentrations. The linear relationships between the absorption and the mass concentration of the pulmonary surfactant-DEPs that follows Lambert Beer’s law can be seen in both LIT and UV-VIS measurements. However, the LIT calibration is more sensitive than UV-VIS calibration, therefore the LIT-based quantitative analysis is more used to support the UV-VIS quantification analysis and to assess the translocation of DEPs at lower concentration (LOD_UV-VIS_ > LOD_LIT_) (Supplementary S2). Taken together, we assessed the translocated fraction of the pulmonary surfactant-DEPs across porous permeable PET membranes with various pore sizes by combining TEM for DEPs visualization with UV-VIS and LIT for DEP quantification. The three techniques are complementary, since the detection and/or visualization of the translocated DEPs fraction is difficult with one of them, it is compensated with the other(s); for instance, the translocated fraction of DEPs was not detected across the PET 0.4 at 2 µg.mL^-1^ DEPs with the UV-VIS technique (0.00%), but it has been detected and visualized by LIT (31%) and TEM, respectively. Likewise for the translocated DEPs fraction across the PET 1.0 at 2 µg.mL^-1^ DEPs, the LIT did not detected it, nevertheless it has been observed by UV-VIS (5%) and TEM. Finally, the translocated fraction of DEPs across PET 0.4 at 20 µg.mL^-1^ DEPs was nor detected by UV-VIS technique, or visualized by TEM, but it has been detected by LIT (11%) (Fig. 3). Thus, the combination of the three methods allow us to confirm with confidence the translocation of DEPs across PET membranes. Interestingly, the percentage of DEPs translocation seems to be concentration depended, since at the lowest applied concentration (2 µg.mL^-1^) the percentage of translocation is significantly higher compared to the middle (20 µg.mL^-1^) and highest concentrations (80 µg.mL^-1^), suggesting the formation of large size agglomerates of DEPs at high concentrations prevent their translocation across the membrane with smaller pores.

### Detection of the translocated fraction of DEPs across the alveolar epithelial type II (A549) cells grown on 3.0 μm porous PET membrane and estimation of the translocation percent of DEPs with UV-VIS and LIT methods

The combination of methods to assess the translocation of DEPs was also applied to an alveolar epithelial tissue model. Other methods to assess the translocation of NPs across a biological barrier have been reported in the past few decades. For instance, Fragioni, Tsuda and colleagues have observed a rapid translocation from the lung airspaces to the body by using near-infrared (NIR) fluorescent NPs [43]. Günter Oberdörster, et al. have studied the translocation of iridium (192)Ir radiolabeled carbon aggregates across the lung of rats to the blood and secondary target organs [22]. However, none of them used physiologically modified DEPs upon biological fluids interaction to investigate their translocation percent across tissues. Since any changes of the DEPs will influence their interactions with cells, it is important to mimic the microenvironment of the DEPs in the alveoli region prior exposure to have the same physicochemical characteristics [44]. Therefore, the DEPs were first prepared in dispersion medium containing pulmonary surfactant prior the cell exposure, and none labelling steps were carried-out. The highest dose that was applied to the A549 was 80 µg.mL^-1^ for 4 h, then post-exposed to the deposited fraction of DEPs for 20 h. The presence of DEPs in the tissue does not induce any cytotoxic effects and does not affect the tight junction organization between the polarized alveolar epithelial cells. However, important releases of IL-8 and IL-1b were observed in the basal fraction (Supplementary S5), indicating an acute inflammatory mediator release in the basal fraction and can potentially affect the organs beyond the lung tissues. The percentage of translocation of DEPs across the alveolar epithelial barrier showed with both UV-VIS and LIT techniques a low amount of translocation, i.e. < 1%. This finding is in line with *Günter Oberdörster et al.* where they discover the percent of translocation of inhaled iridium and carbon nanoparticles aggregates across the lungs of rats less than 1%. *Manuela Semmler-Behnke et al.* has also shown a similar rate of translocation with AuNPs across mice lung tissue (0.5% for AuNPs 200 nm). Interestingly, a low rate of AuNPs has been also reported across alveolar epithelial in vitro tissue [22, 45, 46]. Thus, the current in vivo and in vitro findings correlate with our methodical approach to assess translocation of particles across a biological in vitro tissue. After EDTA treatment, the opening of the paracellular route by losing the tight junction between the cells, we observe a significant increase in the translocation of DEPs across the cells with all methods (UV-VIS, LIT, and TEM), highlighting the importance of the tight junction which are responsible for the tissue integrity and prevent from particles translocation. Furthermore, our data showed that at lower concentration of DEPs, we have a smaller size of agglomerates and a higher percentage of DEPs translocation. Since carbonaceous materials are rather amorphous in aqueous dispersion, the size of DEPs agglomerates increases with the dose [47]. Additionally, the size dependent translocation has already been demonstrated with Wolfgang J. Parak and Manuela Semmler-Behnke, et al. in mice upon AuNPs instillation [46]. Together, our finding indicates that the translocation of DEPs can be potentially tuned by adjusting the size of the agglomerates which also depends on the applied concentration of DEPs. This study on the translocation percentage of DEPs is crucial since in our daily life, we inhaled combustion-derived particles and most of them are small enough to reach the blood circulation and accumulate on the secondary tissues such as the liver, the urinary bladder, and the gastrointestinal tissues. Their impact on those organs beyond the lungs are poorly understood [21, 43, 45, 46].

We propose in this study a combination of methods that have been optimized and can be applied to various combustion-derived particles from different sources, typically for ultrafine particles (UFPs) in order to understand the aerosol toxicity in organs beyond the lung. The methodical approach can help to verify the hypothesis of the trojan horse effect of translocated UFPs that may act as nanocarriers that have adsorbed chemicals such as metal/metal oxides and PAHs upon condensation and then released them in the blood circulation or/and lymphatic vessels to affect additional tissues such as brain, intestinal, liver, as well urinary bladder.

## Conclusion

We propose a combination method to assess the translocation of combustion-derived particles, i.e., DEP, across a biological in vitro tissue by combining qualitative (with TEM) and quantitative (with UV-VIS and LIT) approaches. The choice of the techniques was based on the black carbon core of traffic-related particles, a common physical characteristic of combustion-derived particles. Therefore, the procedure could be applied to any traffic-generated particles, thus enabling to understand their effects to our health. In addition, we provide evidence that a low fraction of DEPs can cross the alveolar epithelial tissue, suggesting the potential systemic effects, especially under chronic exposures, linked to traffic-borne particles accumulation in secondary organs. The nature and origin of the potential mediators that are released in the blood and lymphatic circulations, and inducing the highest toxicity to the secondary organs could be also investigated. Although the translocation of particles may be low, it should be noted that the experiments were carried out following a single exposure at high doses, whereas in real life exposure is in long-term with low doses and can lead to accumulation in organs beyond lungs with an inefficient clearance.

## Methods

### DEP suspension preparation

The SRM2975 referring to standardized diesel exhaust particles (DEPs) was provided by the National Institute of Standards and Technology (NIST, U.S.) and used in this study. DEP suspensions at 2.56 mg.mL^-1^ were prepared according to the NANOGENOTOX dispersion protocol with a slight difference in the dispersion medium (0.05% w/v BSA-water), which was replaced by a sterile Milli-Q water (MQ water) or by the 0.05% w/v CUROSURF® (Chiesi Farmaceutici S.p.A., Parma, Italy), a lipid-based surfactant from pig, in-water dispersion medium (MQ-Curosurf) [48–50]. DEPs suspensions were then sonicated with a horn-probe sonicator (Branson SFX550 Sonifier, U.S.) equipped with a standard 13 mm diameter disruptor horn (Branson Ultrasonics Corp, U.S.) at the delivered acoustic power suggested in the European SOP [51]. DEP stock solutions were further diluted in sterile MQ water at 0.1, 2, 20, and 80 µg.mL^-1^ for DEPs characterization (MQ-water and MQ-Curosurf-based DEPs), and in a sterile phenol-red free cell culture medium (CCM, Minimum Essential Medium, Cat No. 51,200,038, Gibco, ThermoFisher Scientific, Switzerland) at 2, 20, 40, and 80 µg.mL^-1^ for testing DEP translocation (CCM and CCM-Curosurf based DEPs). Prior to experiments DEP solutions were always mixed for 30 s and then sonicated for 5 min at 80 kHz with the maximal power of the bath sonicator (Elmasonic P 60 H, ELMA, Germany).

### Characterization of DEPs solutions

#### Sedimentation profile of DEPs in solution

Five milliliters (mL) of the diluted DEPs in MQ water, MQ-Curosurf, CCM, and CCM-Curosurf at 2, 20, and 80 µg.mL^-1^ were sonicated for 5 min at 80 kHz with the maximal power of the bath sonicator to be well-mixed in 15 mL conical falcon tubes. Thereafter, the prepared DEP solutions were placed in the incubator at 37 °C, in 5% CO_2,_ and the sedimentation state of DEPs in solutions was monitored visually during 24-h incubation. The sonication can change the properties of the particles. However, to establish the methods for assessing DEP translocation, this is a valuable method to minimize the aggregation of DEPs under controlled conditions.

#### Dynamic light scattering (DLS), zeta potential (ζ), and pH analysis of DEPs in solution

To investigate the physical characteristics and the surface charge of DEPs in solutions, the hydrodynamic diameter ${\text{D}}_{\text{h}}$, polydispersity PDI, and zeta potential ζ of DEPs in MQ water, MQ-Curosurf, CCM, and CCM-Curosurf at 20 µg.mL^-1^ were measured using a Particle Size Analyzer (Brookhaven 90Plus, Brookhaven Instruments, Holtsville, USA) with the phase-amplitude light scattering (PALS, Brookhaven Instruments, Holtsville, USA) for zeta-potential determination (at an angle of 90° with a 40 mW diode laser, λ = 635 nm). The pH of each DEPs solution was also measured with the pH meter (827 pH lab, Metrohm, Switzerland).

### Visualization of DEPs with darkfield enhanced-hyperspectral imaging (DF-HSI) and transmission electron microscopy (TEM)

To visualize DEPs in solutions, the MQ-Curosurf-based DEPs solutions from 0.1 to 80 µg.mL^-1^ were dried on microscope glass slides mounting with a cover slip for darkfield enhanced-hyperspectral imaging (DF-HSI) (Cytoviva Inc., Auburn, United States) and on 300 mesh carbon membrane-coated copper grids for transmission electron microscopy (TEM) (Cat No. 71,150, Electron Microscopy Sciences, U.S.). The hyperspectral data cube was achieved using a spectrophotometer (400–1000 nm) and recorded on a Pixelfly camera (PCO AG, Kelheim, Germany). The TEM imaging was carried out on a FEI Tecnai Spirit operating at a voltage of 120 kV and equipped with a side-mounted Veleta CCD camera (Olympus). The micrograph images were processed with Image J (Fiji software, NIH, USA).

### Detection of DEPs with Ultraviolet-visible spectroscopy (UV-VIS) and lock-in thermography (LIT)

The behavior of DEPs in MQ water, MQ-Curosurf, CCM, and CCM-Curosurf at 20 µg.mL^-1^ were monitored by the ultraviolet-visible (UV-VIS) spectrophotometer (V-670 UV-Vis-NIR, Jasco, Japan) at 748 nm wavelength [52]. Furthermore, to quantify the DEPs fraction in solution, Lambert-Beer’s law was verified with a series of dilutions of DEPs in MQ-Curosurf and in CCM-Curosurf in the range of the concentrations from 0.1 to 80 µg.mL^-1^. In brief, one 1 mL of the diluted DEPs samples was added in a 10 mm path-length disposable UV cuvette (Sarstedt, ref 67.742, Germany) for extinction spectrum measurement by the UV-VIS-NIR spectrophotometer. MQ-water was used as a reference for background correction. Accordingly, the correlation between DEPs concentrations and their light extinction was established. To reinforce the reliability and accuracy of the method two independent replicates were employed for this technique.

Due to the thermally conductive characteristics of DEPs, lock-in thermography (LIT) as previously described [53, 54] was also used to quantify the DEPs concentration in MQ-Curosurf. Briefly, 100 µL of the diluted DEPs samples in MQ-Curosurf ranging from 0.1 to 80 µg.mL^-1^ were dried on cell culture Petri dishes (ClearLine, ref 131,046 C, France) for LIT measurement using 525 nm LED light with a stimulation frequency of 0.5 Hz and current of 150 mA (power: 45.3 kW). The diameters of dried samples were measured. The duration of a single measurement was set to 20 s (i.e., 10 cycles) for each sample, and repeated three times consecutively (in total 60 s per sample). The generated amplitude images were further evaluated by using Python programming (Colab Fribourg, Switzerland) to extract the mean amplitude of the thermal signal over the sample area. The calibration curve of the DEPs fraction in MQ-Curosurf was obtained by plotting the mean amplitude of the thermal signal against the known mass over the sample area of dilutions. For reliability and accuracy, three independent replicates were used for this technique.

### Translocation analysis of DEPs across permeable cell culture inserts

To assess the translocation of the DEPs across permeable cell culture inserts, 500 µl of DEPs in MQ-Curosurf at 2, 20, and 80 µg.mL^-1^ (1.1, 11, and 44 µg.cm^-2^) were added to 12-well cell culture inserts (Corning, U.S.) with three pore sizes i.e., 0.4, 1.0, and 3.0 μm of permeable polyethylene terephthalate (PET) membranes that were placed in the 12-well plates (Corning, U.S.) with 1.5 mL of CCM on the basal side. Samples were incubated in an incubator at 37 °C, in 5% CO_2_ and 95% humidity for 24 h and then the basal CCM was collected into the sterile 2 mL Eppendorf tubes for centrifugation at 4 °C for 20 min (16,900 rcf, Centrifuge 5418 R, Eppendorf, Germany). After centrifugation, the obtained pellets containing DEPs were washed with sterile MQ water, re-centrifuged at 16,900 rcf, 4 °C for 20 min, and the pellets were resuspended in 1 mL of MQ water for further analysis.

### Qualitative and quantitative analysis of the basal DEP fractions

The fractions of resuspended DEPs in 1 mL MQ water were first mixed for 30 s and sonicated for 5 min with the bath sonicator, then transferred in 10 mm path-length disposable UV cuvettes for measuring the absorbance of the translocated fractions of DEPs in the basal fraction using the JASCO V-670 UV-VIS-NIR spectrophotometer at 748 nm wavelength. The measured absorbances were converted into mass-based concentrations by using the calibration curves that were previously fitted to determine the percentage of translocation of DEPs across the PET membranes with various pore sizes.

The fraction of DEPs from the 10 mm path-length disposable UV cuvettes were collected again in new sterile Eppendorf tubes and centrifuged at 16,900 rcf, for 20 min at 4 °C. The pellets were resuspended this time in 200 µL of MQ water. After mixing and sonicating, 100 µL of the samples were drop-casted and dried onto Petri dishes. Subsequently, the dried samples were measured with LIT. The mean amplitudes of the thermal signal over the sample area were calculated and then converted into mass/surface ratio to quantify the translocated fractions of DEPs in the basal fraction by Python programming. The remaining 100 µL of the samples were used for qualitative analysis with the TEM imaging as described in the previous section.

### Translocation analysis of DEPs across human alveolar epithelial tissue A549 barrier model

#### Cell culture and DEPs exposure

Materials for cell culture were bought from Gibco, ThermoFisher Scientific (Switzerland). Human alveolar epithelial type II cells (A549 cell line) from American Tissue Type Culture Collection (ATCC) were seeded onto PET membrane inserts with a pore size of 3.0 μm at a density of 277’778 cells.cm^-2^ [55]. Cells were cultured with completed RPMI Medium 1640, 10% [v/v] heat-inactivated FBS, 1% [v/v] L-glutamine, and 1% [v/v] penicillin-streptomycin for four days at 37 °C, in 5% CO_2_ and 95% humidity to reach confluence. On day 5, the A549 cells were set into the air-liquid interface for 24 h. On day 6, A549 cells were washed twice with phosphate-buffered saline (PBS) solution and apically exposed to 500 µL of DEPs in CCM-Curosurf at 20, 40, and 80 µg.mL^-1^ (11, 22, and 44 µg.cm^-2^). Fresh medium (1.5 mL) was added to the basal compartments. A549 cells were exposed to DEPs for 4 h at 37 °C in 5% CO_2_ and 95% humidity; to avoid dose limitations and big agglomerates, the cells were exposed to DEPs under submerged conditions. Subsequently, the apical fraction was removed, and the cells were cultured at the ALI conditions for an additional 20 h, then the conditioned basal fractions were collected in the sterile 2mL Eppendorf tubes for UV-VIS, LIT, and TEM analysis. For control experiments, the cells were exposed in parallel to 5 mM ethylenediaminetetraacetic acid (EDTA) and DEPs. The exposure of cells to 5 mM EDTA alone does not influence the cell viability.

### DEPs deposition on A549 cells with LIT

After 24-h incubation the alveolar epithelial tissue was washed twice with PBS and then fixed with 4% of paraformaldehyde (PFA) in PBS for 15 min. Thereafter, the fixative was discarded, and the cells were washed twice with the PBS solution. The fixed sample on PET membrane was cut out from the inserts with tweezer and scalpel. The membrane was placed on a Petri dish and dried in a desiccator overnight. Later on, the dried sample was measured with the LIT.

### Transepithelial electrical resistance (TEER), lactate dehydrogenase (LDH), and enzyme-linked immuno assay (ELISA) analysis

After 24-h incubation, 500 µL of RPMI medium was added to the apical compartment of the inserts. Subsequently, the apical and basal media were collected in Eppendorf tubes to assess the cytotoxicity and inflammatory conditions of cells. To evaluate the epithelial integrity, the transepithelial electrical resistance (TEER) value of cells was measured by adding 500 µL of fresh RPMI medium to the apical and 1.5 mL to the basal compartments of inserts before and after DEP exposure using a Millicell® ERS-2 (Electrical Resistance System, Millipore, Switzerland) equipped with an STX01 electrode (World Precision Instruments, Switzerland). Prior to each measurement, the electrodes were sterilized in 70% ethanol and then rinsed with sterile 1x PBS. TEER values were presented after the correction for the insert area (0.9 cm^2^) and the resistance of cell-free 12-well inserts.

To assess the cytotoxicity and inflammation, the collected media were centrifuged at 500 rcf, 4 °C for 5 min to remove cell debris. Subsequently, 100 µL of the apical and basal fractions were used from the Eppendorf tubes to investigate the release of lactate dehydrogenase (LDH) into the supernatants as a result of cell membrane rupture using a commercially available LDH kit (Roche Applied Science, Germany). The enzymatic activity of collected samples was measured photometrically at an absorbance of 490 nm with a reference wavelength of 630 nm. LDH values are presented relative to the positive control (cells treated with 0.2% Triton-X-100 apically for 12 min). CCM-Curosurf was used as a negative control. Similarly, 100 µL of the apical and basal fractions were used to quantify the release of inflammatory mediators such as IL-8, IL-6, IL-1b, and TNFα in the medium using a commercially available sandwich ELISA protocol (R&D Systems DuoSet®, US). In this assay, a capture and detection antibodies recognized specifically a mediator; the detection antibody was conjugated to streptavidin-horseradish peroxidase (HRP) enzyme which reacted with substrate solution; the colorimetric reaction was stopped after 20 min of incubation at room temperature, and the absorbance of the final solution was measured at an absorbance of 450 nm with a wavelength correction at 570 nm. A linear standard calibration curve was also plotted to quantify the amount of the mediator that was released in the medium for each condition. The exposure of cells to LPS or TNFα was used as a positive control.

### Confocal laser scanning microscopy (cLSM) imaging of deposited fraction of DEPs on A549 cells

Following the 24-h incubation, the cells were washed twice with PBS, then fixed with 4% PFA (w/v), and again rinsed twice with PBS to eliminate the remaining PFA. Subsequently, the cells were washed with 0.1% BSA in PBS and then incubated in 0.2% Triton X-100 with PBS for 15 min to allow permeabilization of the cell membrane. Then, the cells were incubated with 1% BSA in PBS for 1 h at room temperature before the staining to reduce unspecific bindings. Next, for nuclei and cytoskeletal staining, 4′,6′-diamidino-2-phenylindole (DAPI, 1:50 dilution; stock concentration 100 µg/mL) and Alexa Fluor™ 488 phalloidin (1:100 dilution; Life technologies, Cat. No A12379) in 0.1% BSA were respectively added to the cells for 2 h. Thereafter, the cells were washed three times with PBS and mounted for immunofluorescence imaging. Visualization was done using a Leica Stellaris 5 confocal LSM (Leica Microsystems, Wetzlar, Germany) at excitation wavelengths λex = 405 nm and λex = 488 nm sequentially scanned. The image processing was done using ImageJ (NIH, Bethesda, Maryland, USA).

### Data analysis

All data are presented as mean ± standard deviation. A total of three independent experiments (*n* = 3) were performed for the DEPs characterization in solution, UV-VIS, LIT, TEER, and LDH measurements. Statistical analysis was performed using GraphPad Prism (GraphPad Software Inc., la Jolla, United States). Assuming a normal distribution of the data sets, a parametric unpaired t-test for particles characterization in solution and one-way analysis of variance (ANOVA) for UV-VIS, LIT, TEER, and LDH measurements were performed for statistical comparisons with a significance level of *p* < 0.05.

### Electronic supplementary material

Below is the link to the electronic supplementary material.

Supplementary Material 1

## Data Availability

No datasets were generated or analysed during the current study.

## Abbreviations

DEPs: Diesel exhaust particles
PM: Particulate matter
UFPs: Ultrafine particles
PAHs: Polycyclic aromatic hydrocarbons
IARC: International agency for research on cancer
AuNPs: Gold nanoparticles
MQ: MilliQ
CCM: Cell culture medium
DLS: Dynamic light scattering
PDI: Polydispersity index
D_h_: Hydrodynamic diameter
ƺ: Zeta potential
DLVO: Derjaguin-Landau-Verwey-Overbeek
SMPS: Scanning mobility particle sizer
SEM: Scanning electron microscopy
TEM: Transmission electron microscopy
cLSM: Confocal laser scanning microscopy
EDF-HSI: enhanced darkfield hyperspectral image
PET: Polyethylene terephthalate
ALI: Air-liquid interface
TEER: Transepithelial electrical resistance
LDH: Lactate dehydrogenase
EDTA: Ethylenediaminetetraacetic acid
UV-VIS: Ultraviolet-visible
LIT: Lock-in thermography
NIR: Near infrared
LOD: Limit of detection
IL-8: Interleukin-8
IL1β: Interleukin-1β
IL-6: Interleukin-6
TNFα: Tumor necrosis factorα
