# Supplementary material for: Combining analytical techniques to assess the translocation of diesel particles across an alveolar tissue barrier in vitro
Source: Part Fibre Toxicol. 2024 May 22;21:26. doi: 10.1186/s12989-024-00585-7 (PMC11110323; doi:10.1186/s12989-024-00585-7)

S1.


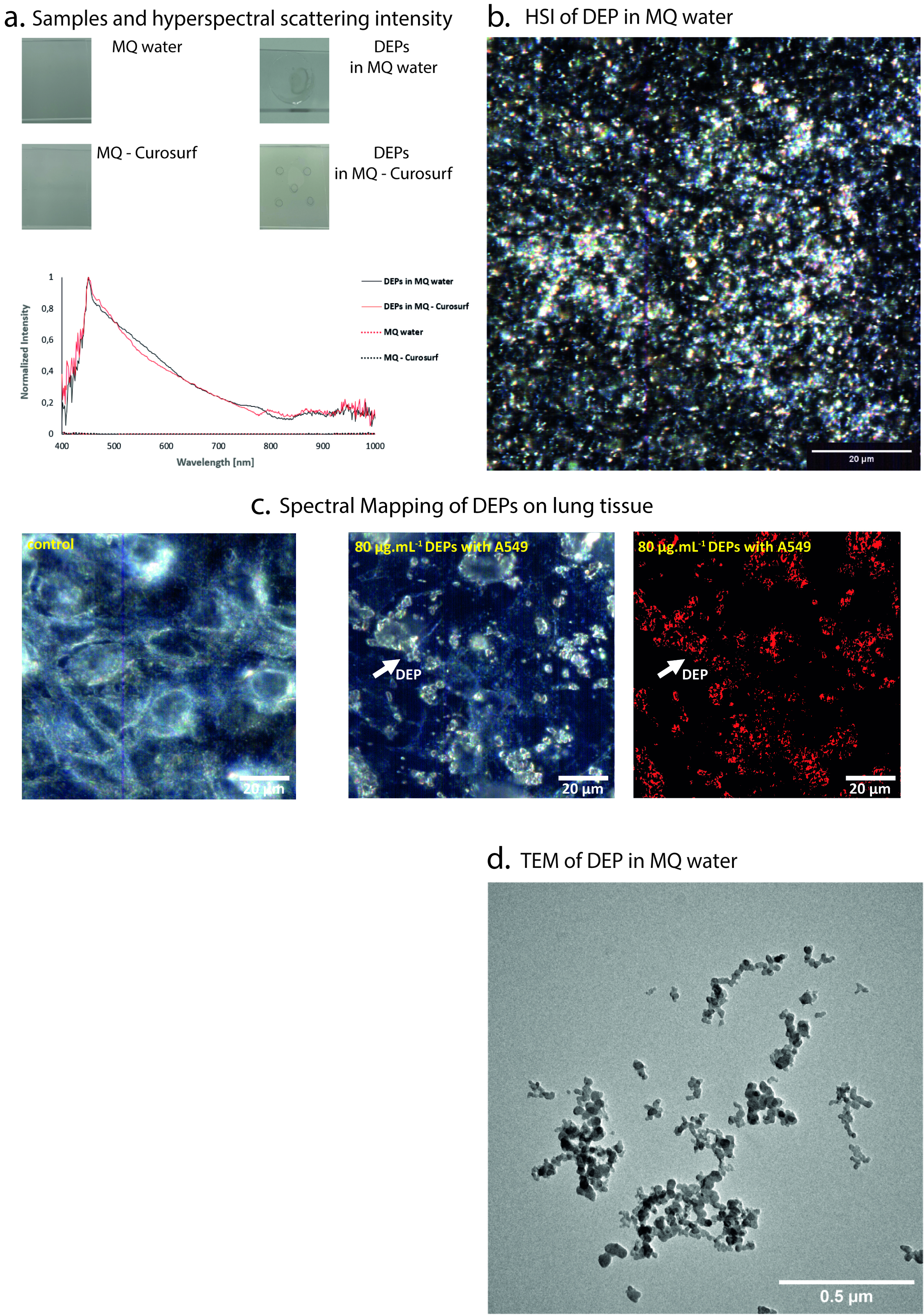

**Supplementary S1. Visualization of dried DEPs from MQ water and MQ-Curosurf solution at 80 µg.mL^-1^.** **a.** The hyperspectral scattering intensities of DEP in MQ water and in MQ-Curosurf are displayed. The controls correspond to MQ water and MQ-Curosurf solution without DEPs. **b.** The hyperspectral image (HSI) of DEPs in MQ water, scale bar: 20 µm. **c.** Spectral mapping of DEPs on A549 cells, scale bar: 20 µm . **d.** The transmission electron microscopy on DEPs in MQ water, scale bar: 0.5 µm.

S2.


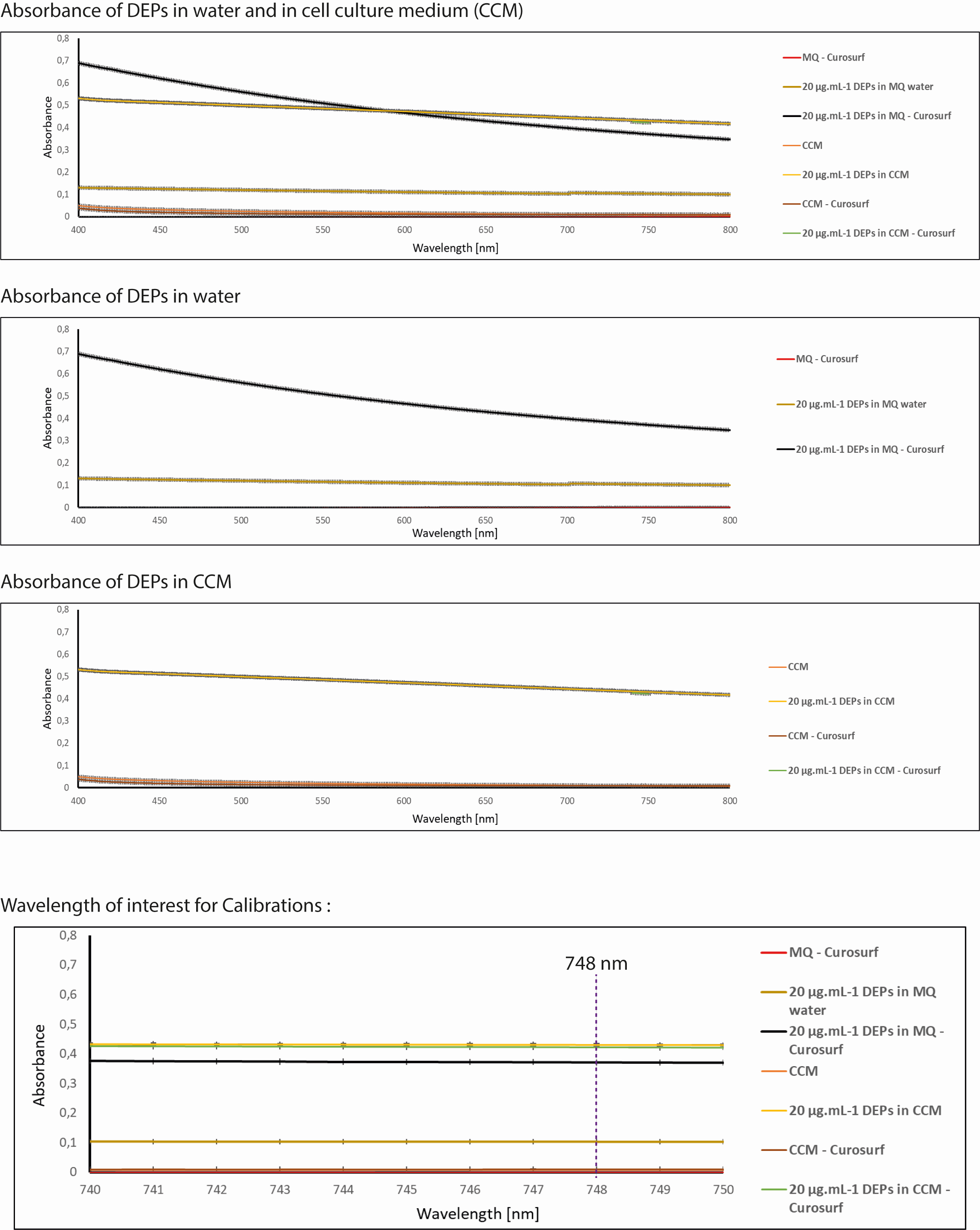


a.


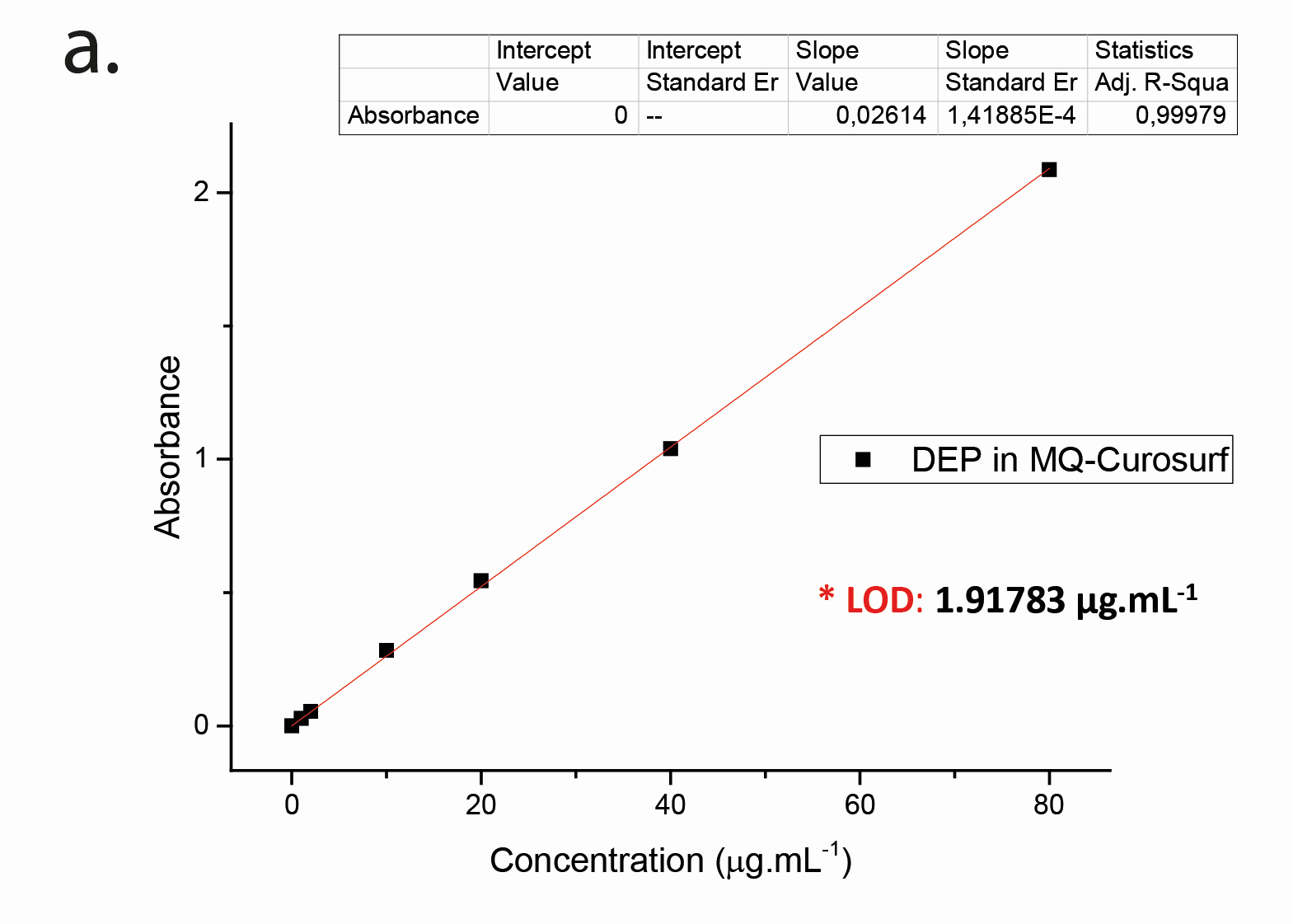


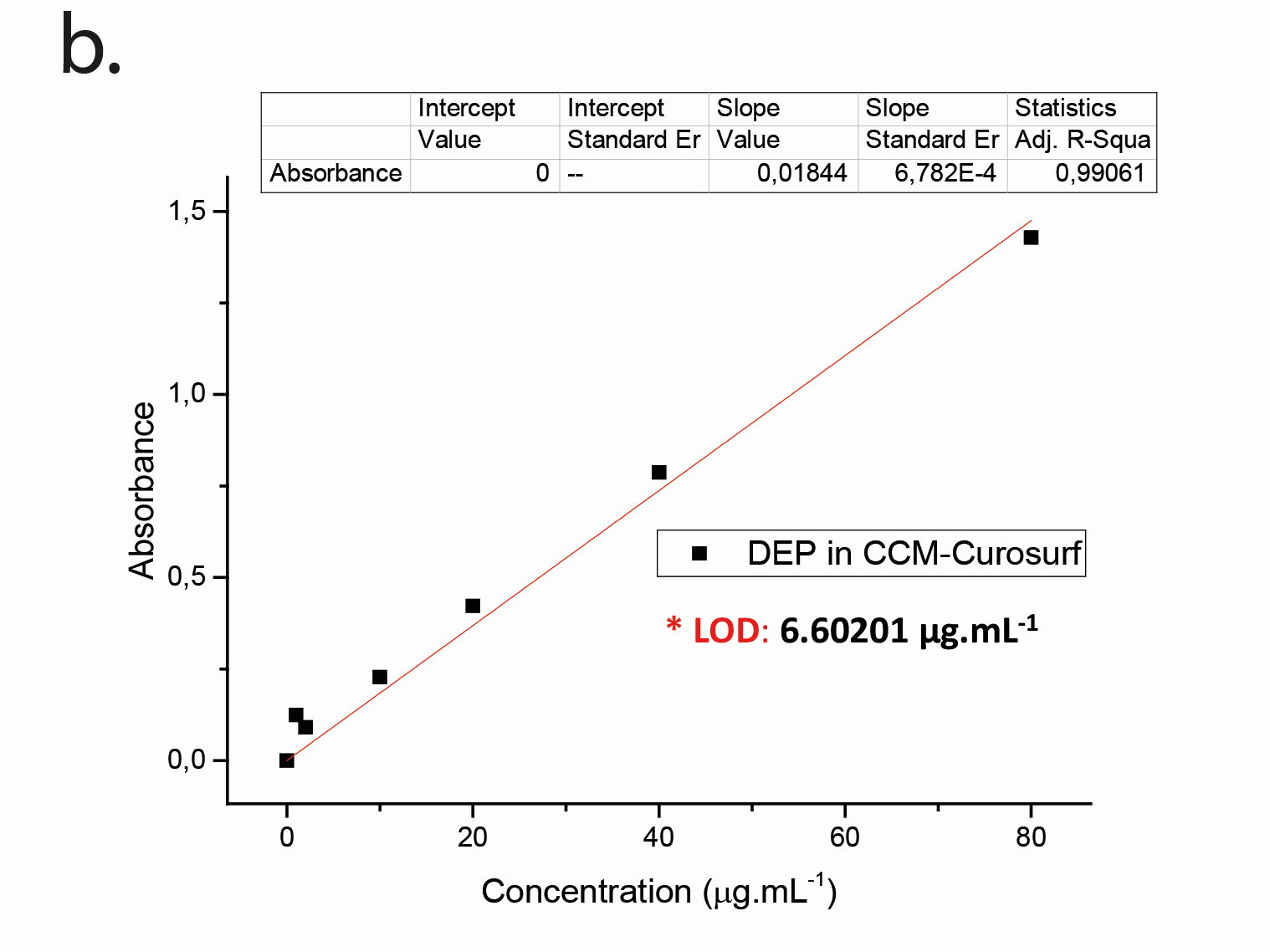


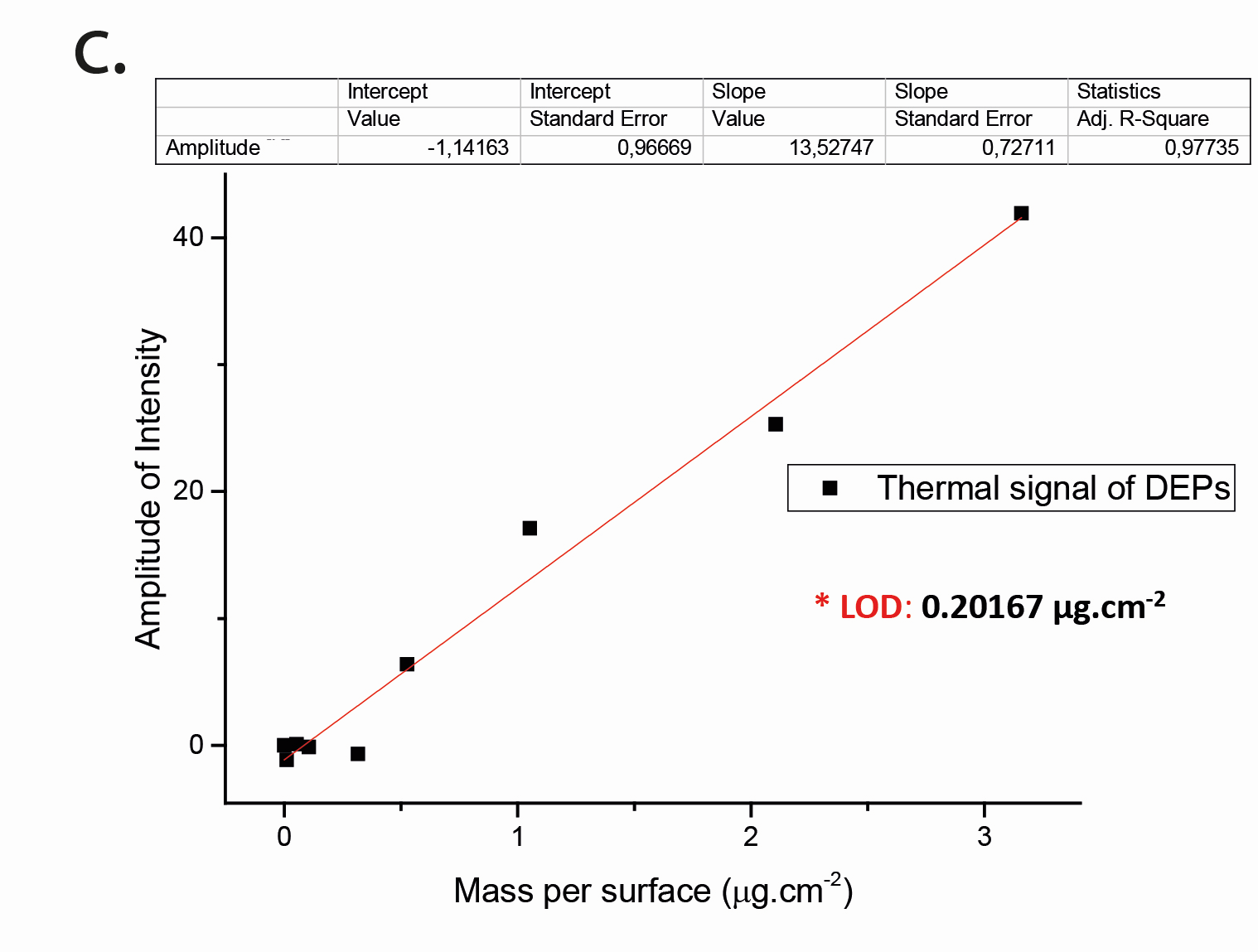


b.

c.

**Supplementary S2**. **a.** Absorbance spectroscopy of DEPs in MQ water and CCM. **b. and c.** Linear calibration with UV-VIS absorbance spectroscopy between DEPs absorbance and DEPs mass concentration in MQ-Curosurf (a) and in CCM-Curosurf solution (b). **c.** Linear calibration with LIT between DEPs thermal signal and the DEPs mass concentration in MQ-Curosurf solution. The means ± SD are from two independent experiments performed in triplicates (N=3). The LOD represents the limit of detection of the DEPs.

S3.


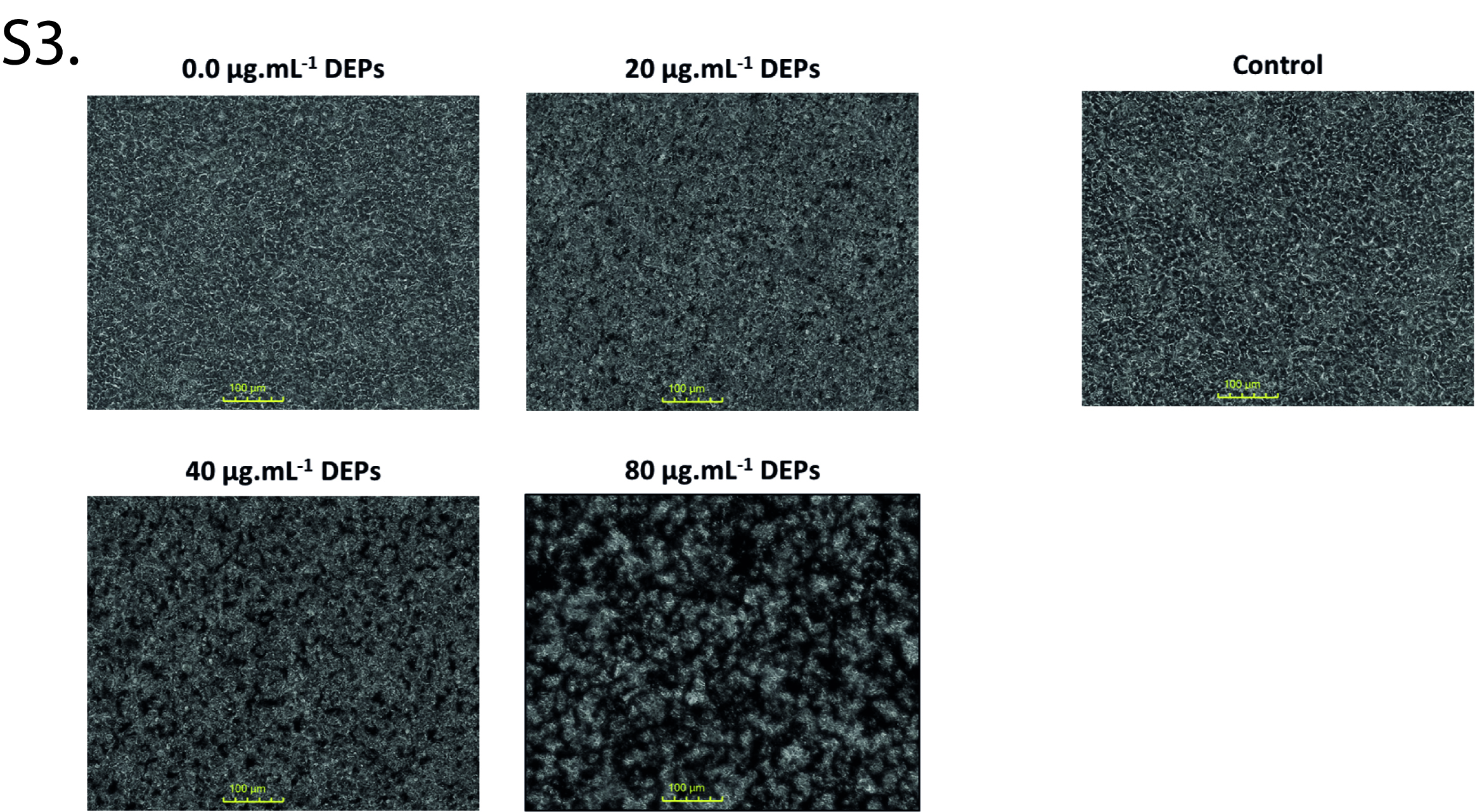


**Supplementary S3**. Phase contrast images of deposited DEPs at 20, 40, and 80 µg.mL^-1^ on the A549 monolayer.

S4.

**Supplementary S4**. Thermal emission of MQ water, MQ - Curosurf, CCM, and CCM - Curosurf from 525nm LED wavelength as the heating source.


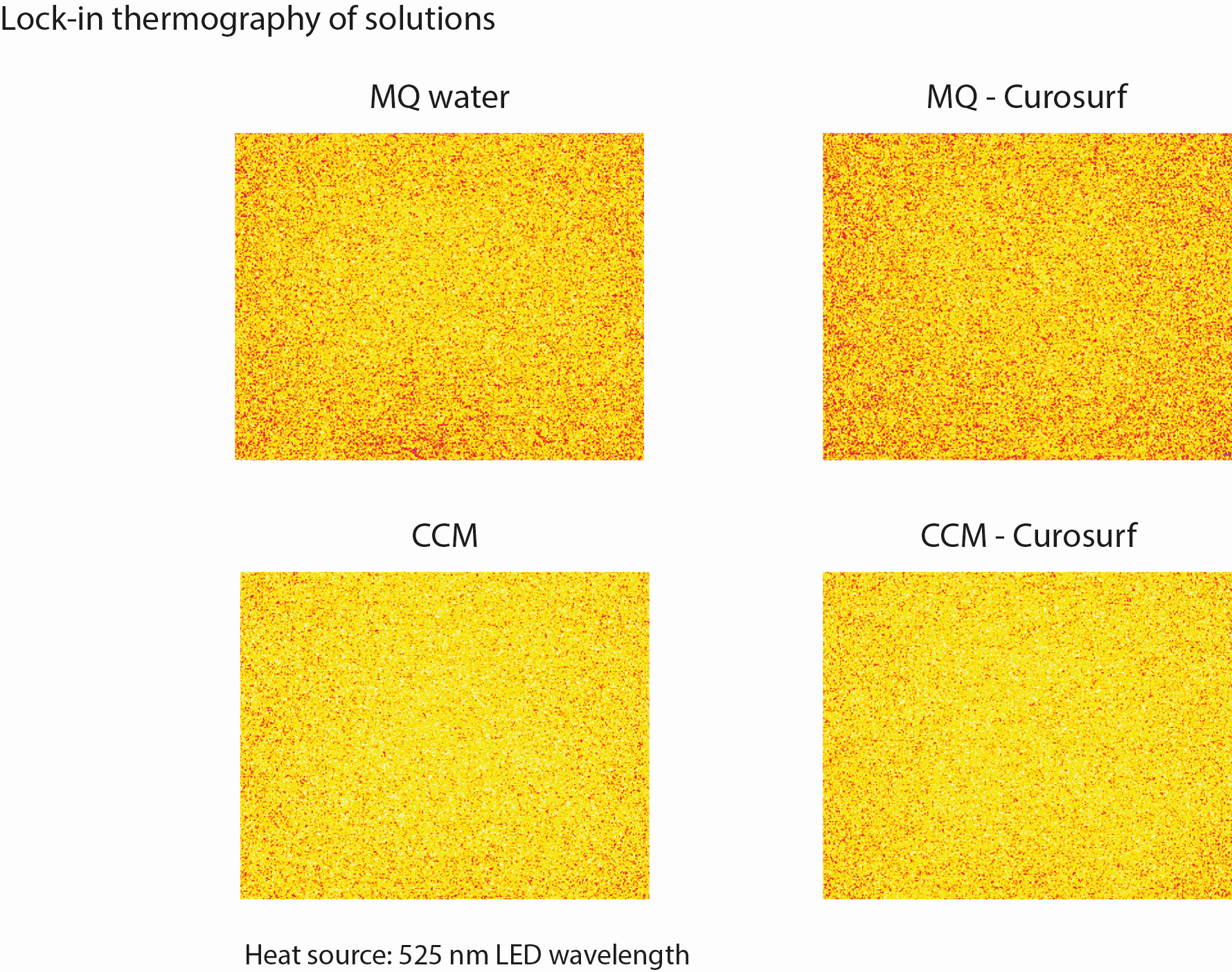


S5.

**Supplementary S5**. Biological mediators IL-8, IL-6, IL-1β, and TNFα release in the basal fraction upon the A549 monolayer exposure to DEPs at 20, 40 and 80 µg.mL^-1^.


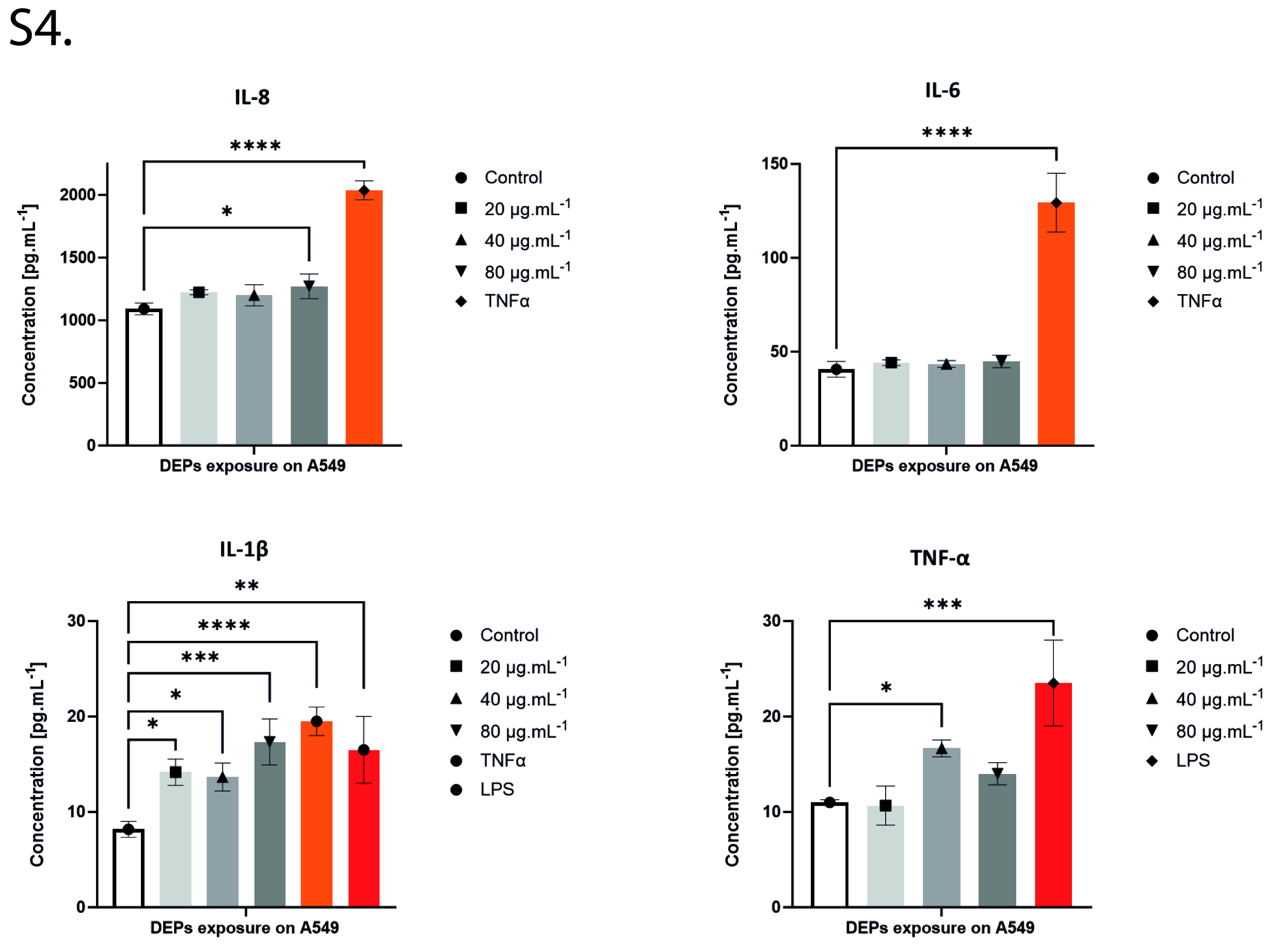

Supplement: Supplementary file 1 — Supplementary Material 1 [file 12989_2024_585_MOESM1_ESM.docx]
